# Total stricture of liver transplant biliary anastomosis resolved with peroral digital cholangioscopy and the non-flexible end of a 0.018-inch biliary guidewire

**DOI:** 10.1055/a-2092-0246

**Published:** 2023-06-12

**Authors:** Maria Puigcerver-Mas, Albert Garcia-Sumalla, Josefina Lopez-Dominguez, Laura Llado Garriga, Joan B. Gornals

**Affiliations:** 1 Endoscopy Unit, Department of Digestive Diseases, Hospital Universitari de Bellvitge, Barcelona, Catalonia, Spain; 2 Bellvitge Biomedical Research Institute (IDIBELL), Barcelona, Catalonia, Spain; 3 Universitat de Barcelona, Barcelona, Catalonia, Spain; 4 Liver transplant Unit, Department of General Digestive Surgery, Hospital Universitari de Bellvitge, Barcelona, Catalonia, Spain

A 64-year-old man underwent orthotopic liver transplantation due to hepatocellular carcinoma on liver cirrhosis diagnosed 2 years earlier. Three months after transplantation surgery, he presented with jaundice, and a cholangio magnetic resonance imaging scan revealed a biliary anastomosis stricture with marked kinking and retrograde dilatation. A multidisciplinary committee decided on an endoscopic approach.

Initially, retrograde biliary cannulation by endoscopic retrograde cholangiopancreatography was attempted, but advancement of different guidewires through the biliary anastomosis was not possible. The use of peroral digital single-operator cholangioscopy helped us to visualize a complex biliary stricture, which explained why it had not been possible to advance any kind of guidewire into the intrahepatic ducts ( Fig. 1 ). A 0.018-inch guidewire (Novagold; Boston Scientific, Marlborough, Massachusetts, USA) was inverted with the purpose of using the non-flexible end as a fine needle. This stiff end was successfully inserted under cholangioscopy/fluoroscopy guidance through the closed anastomosis, similarly to a needle ( Fig. 2 , Fig. 3 ). After this maneuver, the guidewire was restored to its standard position, with the flexible end now able to advance through the anastomosis following the needle-like puncture ( Video 1 ).

**Fig. 1:**
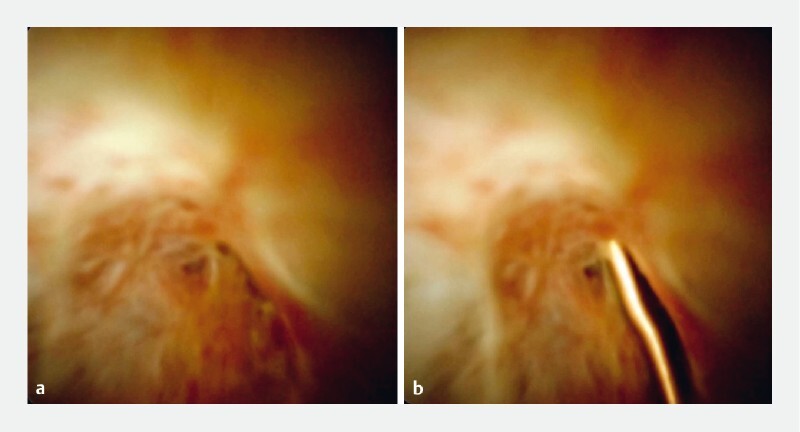
Cholangioscopy images. **a** The post-liver transplant biliary stenosis. **b** The flexible end of a 0.018-inch guidewire pushing against the biliary stricture.

**Fig. 2:**
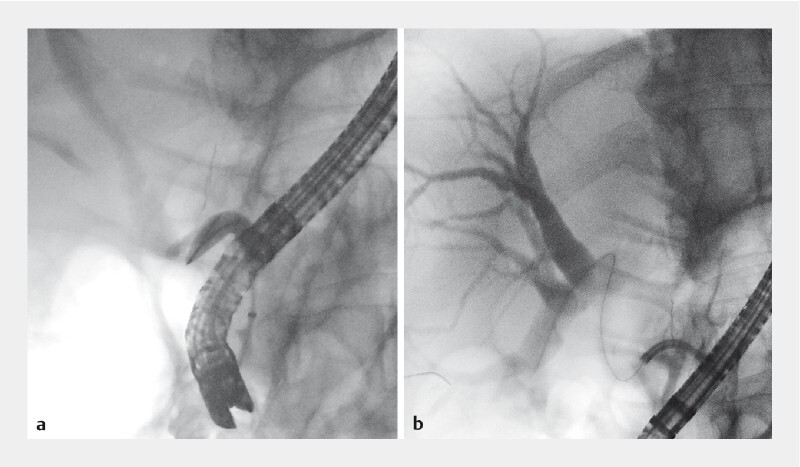
Fluoroscopy images. **a** Failed endoscopic retrograde cholangiopancreatography attempt. **b** Successful use of peroral digital single-operator cholangioscopy with an inverted 0.018-inch guidewire.

**Fig. 3:**
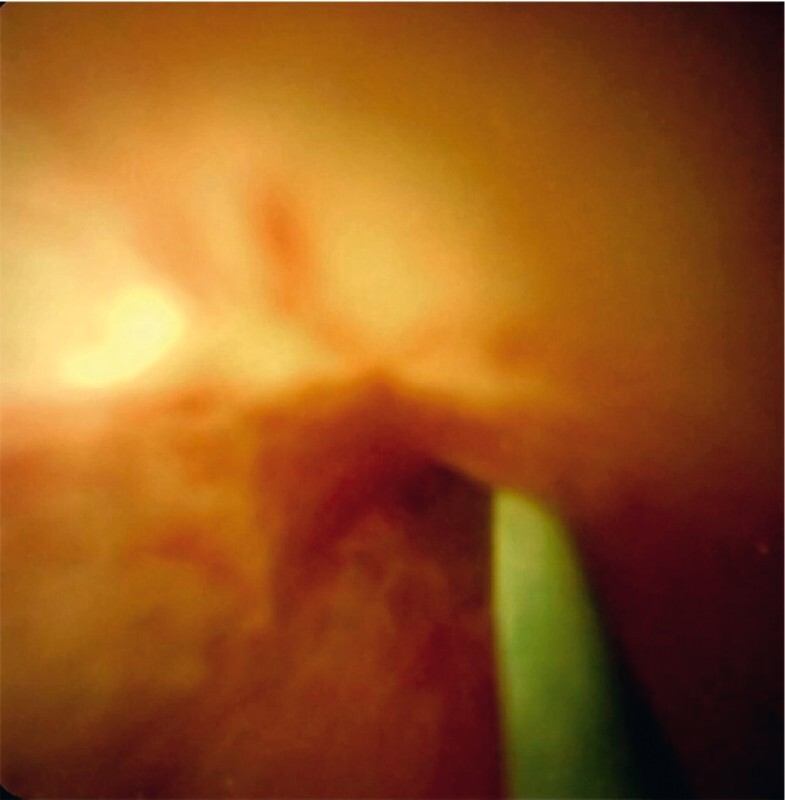
Cholangioscopy image of the non-flexible end of a 0.018-inch guidewire puncturing through the biliary stricture.

**Video 1** Total stricture of liver transplant biliary anastomosis resolved with peroral digital cholangioscopy and the non-flexible end of a 0.018-inch biliary guidewire.


Finally, endoscopic maximal stent therapy (MST) was initiated. First, balloon dilation up to 8 mm was performed, followed by successful placement of three plastic stents (Advanix 10 Fr, 8.5 Fr, and 7 Fr × 12 cm; Boston Scientific) as the first session of a 1-year MST program ( Fig. 4 ).

**Fig. 4:**
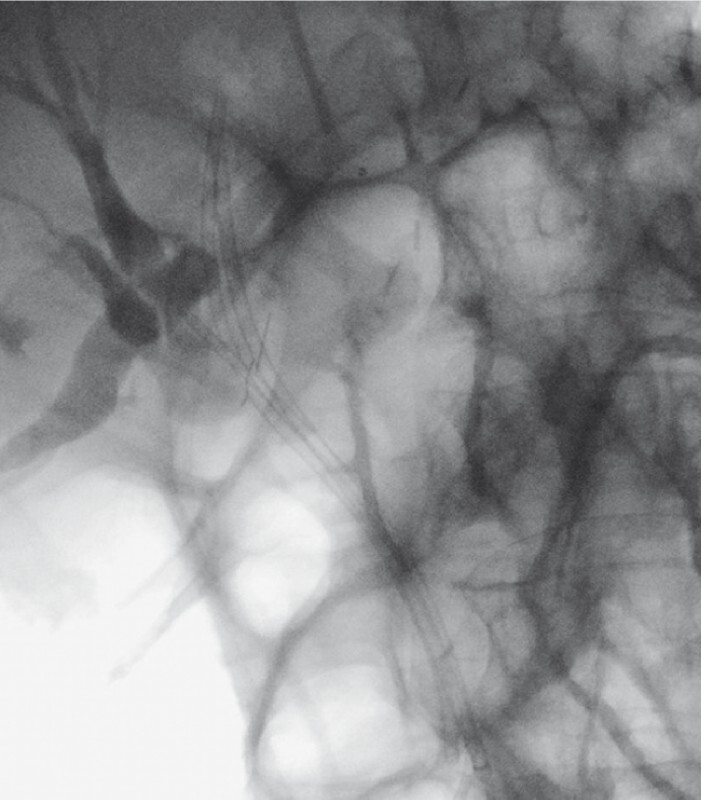
Three biliary plastic stents placed through the biliary stricture, as the first session of a 1-year program of maximal stent therapy.

This case of post-liver transplant biliary stricture was resolved using cholangioscopy/fluoroscopy guidance and a guidewire technical variant. The utility of digital single-operator cholangioscopy for the endoscopic management of biliary strictures after liver transplantation has been published previously 1 2 . However, use of the non-flexible end of a 0.018-inch guidewire as a method of crossing a complete biliary stenosis has not been previously reported.

Endoscopy_UCTN_Code_TTT_1AR_2AZ

## Notes

Endoscopy E-Videos

https://eref.thieme.de/e-videos
*E-Videos* is an open access online section of the journal *Endoscopy* , reporting on interesting cases and new techniques in gastroenterological endoscopy. All papers include a high-quality video and are published with a Creative Commons CC-BY license. Endoscopy E-Videos qualify for HINARI discounts and waivers and eligibility is automatically checked during the submission process. We grant 100% waivers to articles whose corresponding authors are based in Group A countries and 50% waivers to those who are based in Group B countries as classified by Research4Life (see: https://www.research4life.org/access/eligibility/ ). This section has its own submission website at https://mc.manuscriptcentral.com/e-videos
